# Quantifying Individual Variation in the Propensity to Attribute Incentive Salience to Reward Cues

**DOI:** 10.1371/journal.pone.0038987

**Published:** 2012-06-22

**Authors:** Paul J. Meyer, Vedran Lovic, Benjamin T. Saunders, Lindsay M. Yager, Shelly B. Flagel, Jonathan D. Morrow, Terry E. Robinson

**Affiliations:** 1 Department of Psychology, University of Michigan, Ann Arbor, Michigan, United States of America; 2 Department of Psychiatry, University of Michigan, Ann Arbor, Michigan, United States of America; University of Chicago, United States of America

## Abstract

If reward-associated cues acquire the properties of incentive stimuli they can come to powerfully control behavior, and potentially promote maladaptive behavior. Pavlovian incentive stimuli are defined as stimuli that have three fundamental properties: they are attractive, they are themselves desired, and they can spur instrumental actions. We have found, however, that there is considerable individual variation in the extent to which animals attribute Pavlovian incentive motivational properties (“incentive salience”) to reward cues. The purpose of this paper was to develop criteria for identifying and classifying individuals based on their propensity to attribute incentive salience to reward cues. To do this, we conducted a meta-analysis of a large sample of rats (N = 1,878) subjected to a classic Pavlovian conditioning procedure. We then used the propensity of animals to approach a cue predictive of reward (one index of the extent to which the cue was attributed with incentive salience), to characterize two behavioral phenotypes in this population: animals that approached the cue (“sign-trackers”) vs. others that approached the location of reward delivery (“goal-trackers”). This variation in Pavlovian approach behavior predicted other behavioral indices of the propensity to attribute incentive salience to reward cues. Thus, the procedures reported here should be useful for making comparisons across studies and for assessing individual variation in incentive salience attribution in small samples of the population, or even for classifying single animals.

## Introduction

If the impending receipt or availability of a desirable item (a rewarding unconditioned stimulus, US) is signaled by a cue, the cue itself can acquire a number of properties. Best known is the ability to act as a conditioned stimulus (CS), evoking a response (conditioned response; CR) that formerly was elicited only by receipt of the reward itself. Thus, as described by Pavlov [1], a cue that is paired with delivery of food to a hungry dog can come to evoke salivation prior to receipt of the food. However, a Pavlovian cue can also acquire more complex psychological properties. Of particular interest here is the ability of a Pavlovian cue to directly activate emotional and motivational states, influencing behavior via its properties as an incentive stimulus [2], [3], [4], [5], [6]. But, how does one know if a “cold”, informational CS also acquires the properties of a “hot” incentive stimulus, and thus has the ability to incite and motivate actions?

Operationally, Pavlovian incentive stimuli are defined as stimuli that have three fundamental properties [4], [5], [6], [7], [8], [9], [10], [11], [12], [13]. First, incentive stimuli bias attention towards them and are attractive - individuals approach them. This feature of an incentive stimulus will often bring an individual into close proximity with the associated reward. Second, incentive stimuli themselves become objects of desire (‘wanted’), in the sense that individuals will work to get them, and they can even reinforce learning a new instrumental response to get them (i.e., they act as conditioned or secondary reinforcers). This feature of an incentive stimulus can sometimes motivate persistent reward-seeking behavior for long periods of time in the absence of the reward itself. Finally, incentive stimuli can generate a conditioned motivational state that can goad or spur renewed seeking for the associated reward. In the case of drug-associated cues, this feature of an incentive stimulus may produce craving and/or relapse, despite a conscious intent to maintain abstinence. Experimentally, each of these three properties of an incentive stimulus can be assessed using well-established procedures, including Pavlovian conditioned approach (PCA), conditioned reinforcement and Pavlovian-to-instrumental transfer (PIT) or reinstatement procedures.

Importantly, there is considerable individual variation in the extent to which a Pavlovian CS acquires the properties of an incentive stimulus. For example, it has long been known that only some animals come to approach a reward cue [i.e., show what has been called a sign–tracking CR; [14], [15]]. Indeed, Zener [16] described such individual variation seventy-five years ago during experiments in which the ringing of a bell was paired with food delivery in unrestrained dogs. He noted that after learning the association between the CS and US, the topography of the CR varied considerably from animal to animal. Some dogs exhibited a “small but definite movement of approach toward the conditioned stimulus . . . followed by a backing up later to a position to eat”. However, other dogs showed “an initial glance at the bell” followed by “a constant fixation . . . to the food-pan . . .”, and yet others vacillated, looking back and forth between the bell and the food pan. Similar findings were later described in other species, including rats, and these cue- vs. goal location-directed behaviors were termed “sign-tracking” and “goal-tracking” CRs, respectively [15], [17], [18], [19], [20], [21], [22], [23].

We have recently reported that the propensity to approach a food cue also predicts the extent to which the cue acquires other properties of an incentive stimulus. In rats that develop a strong sign-tracking CR (“sign-trackers”; STs), the CS is also a more effective conditioned reinforcer [9], [24], [25] and produces greater reinstatement [26], than in rats that do not approach the CS, but instead learn to approach the food cup (“goal-trackers”; GTs). Furthermore, based on their propensity to approach a food cue, we can predict, prior to any experience with drugs, in which individuals drug cues will come to powerfully control and motivate behavior [27], [28], [29], [30], [31].

These studies have led us to suggest that individual differences in the extent to which food and drug cues acquire the properties of an incentive stimulus reflect an underlying complex psychological trait, which we define as *the propensity to attribute incentive salience to reward cues*. Here, we use the term “psychological trait” to denote a psychological process that has several behavioral manifestations, and “behavioral phenotype” as a measureable manifestation of this psychological trait. Therefore, although the classification of the ST and GT behavioral phenotypes is based on their performance on a Pavlovian approach test, it is important to note that sign- vs. goal-tracking behavior are but one manifestation of the underlying psychological trait, which can be assessed using different tests to determine if a cue is attributed with incentive salience (tests of approach, conditioned reinforcement and reinstatement).

In our previous studies on individual variation in the expression of sign- vs. goal-tracking behavior, we characterized relatively small numbers of rats in any given experiment, using their tendency to sign-track as a phenotypic index of psychological trait variability. However, it is difficult to determine the prevalence and characteristics of such phenotypes in the population using small samples. To better achieve this aim we have pooled data from multiple studies in which animals were tested under nearly identical conditions. Thus, we report here analyses based on data from 1,878 rats tested for conditioned approach using a standard Pavlovian (i.e. “autoshaping”) procedure (see Materials and Methods), providing a good estimate of variation in these behaviors in the population. Our goals were two-fold: (1) to better characterize individual variation in learning a ST CR vs. GT CR, as an index of the propensity to attribute incentive salience to a food cue, and (2) develop a metric to classify and compare individual animals, based on their performance relative to a large sample of the population.

## Results

### Marked individual variation in the propensity to approach a lever-CS vs. the food cup after Pavlovian training in rats

**Figure 1:**
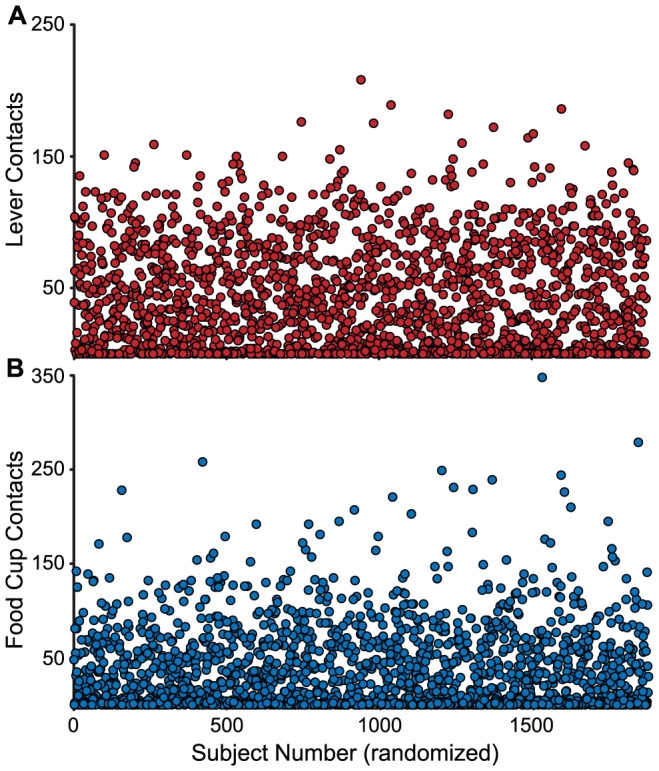
Individual variation in the propensity to approach the lever-CS or food cup after 5 days of Pavlovian training. The number of lever deflections (A) and food cup entries (B) is shown for 1,878 individual rats (Subject Number), with the order of the cases randomized so the values are not clustered. Note that there is enormous individual variation in the preferred response.

Fig. 1 illustrates that there is enormous individual variation in the extent to which rats learn to approach and engage a lever-CS vs. the food cup after 5 sessions of CS-US pairing (Pavlovian training). These figures include data from all rats, before they were classified into behavioral phenotypes as described below. It is readily evident that some animals vigorously engage the lever-CS, whereas others do not. Conversely, some animals approach and engage the food cup during the CS period, and rarely make contact with the lever-CS.

### An Index of Pavlovian Conditioned Approach (PCA) Behavior and Calculation of a PCA Score

In our initial studies, using relatively small samples, we classified animals as STs or GTs using a “rank-order split” method — dividing the sample into thirds based on the absolute number of lever-CS deflections after training [e.g.], [ 9], [32,33]. There are two potential problems with this approach. First, in any small sample the distribution may be skewed towards one phenotype or the other, and simply dividing the sample into thirds may result in misclassifying individuals, and also result in very different performance criteria from one experiment to the next. Second, this way of classifying animals ignores other aspects of conditioned approach, which is a broad term that can be measured in multiple ways. For example, our previous method did not include the degree of interaction with the food-cup (i.e., goal-tracking) when classifying individual rats, even though most animals engage in both sign- and goal-tracking to some extent. Furthermore, some rats may approach the cue or food cup quickly, but not necessarily engage it vigorously, or may respond only on a portion of trials. Because these three aspects of approach may be expressed to different degrees in individual animals, we routinely measure (a) the number of lever deflections and food cup head entries during the CS period, (b) the probability of contacting the lever or entering the food cup during the CS period on each trial (defined as the number of trials with a lever press or food cup entry, divided by the total number of trials), and (c) the latency to the first lever deflection or food cup entry. Using these measures we devised a “PCA Score”, as follows. From the CS lever deflections and CS food cup entries, we calculated three measures of approach: 1) *Response Bias* (ratio of lever presses and food cup entries in relation to total number of responses), 2) *Probability Difference* (the difference between the probability of pressing the lever and the probability of entering the food cup), and 3) *Latency Score* (difference between the latencies to approach the lever and the food cup). Averaging these three measures of approach produces a PCA Score for each individual, on each day of training. Scores derived this way range from −1 to +1, whereby scores of −1 and +1 indicate strong biases toward goal-tracking and sign-tracking, respectively, and a score of zero indicates that the two responses are equally distributed. Table 1 describes these calculations in detail. We also measured the number of food cup head entries during the inter-trial interval (ITI), but these are not included as part of the PCA Score.

**Table 1:** Formulas for deriving the PCA Index Score.

Response Bias	= (Lever Presses – Food Cup Entries)/(Lever Presses+Food Cup Entries)
Probability Difference	= p|Lever Press – p|Cup Entry
Latency Score	= (x̄ Cup Entry Latency – x̄ Lever Press Latency)/8
PCA Score(n)	= [Response Bias(n)+Latency Score(n)+Probability Difference(n)]/3
PCA Index Score	= [PCA Score (4)+PCA Score (5)]/2

(n) = any particular test session.

x̄ = averaged Latency.

p| = probability.

Legend: The overall PCA Index Score, used for phenotype classification (see Fig. 6), was derived by averaging the individual PCA Scores for Days 4 and 5 of training. The PCA Score for each session was derived by averaging the three individual measures (Response Bias, Probability Difference, and Latency Score) for that particular session. Responses Bias is a proportion of lever presses/food cup entries in relation to the total number of responses. The Probability Difference was derived by subtracting the probability of food cup entries from the probability of lever presses. Latency score was the (averaged) difference between latencies to make food cup and lever responses (divided by the length of the CS duration; in this case 8 s).

**Figure 2:**
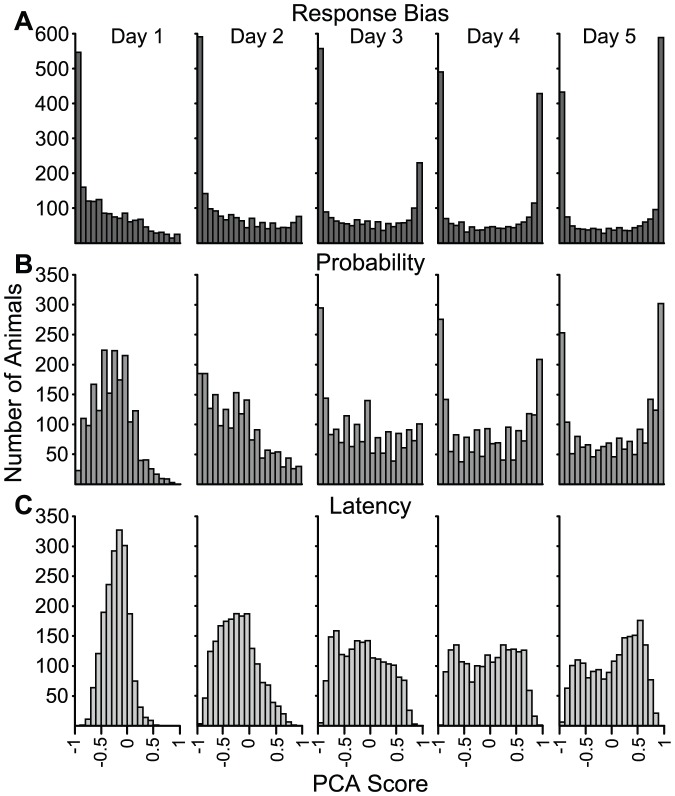
The distribution of each of the three components of the PCA Score across each of the 5 days of Pavlovian training. Panel A shows the Response Bias score, Panel B the Probability Difference score and Panel C the Latency score. The number of rats with a given PCA Score are binned into 20 bins of equal size (0.1 bin sizes), according to their score, which ranges from +1 to −1. Thus, the vertical axis shows the number of rats in each bin, and the horizontal axis the PCA Score. Note that the Response Bias score and the Probability Difference score show the development of two subpopulations by Days 4 and 5 of training.

**Figure 3:**
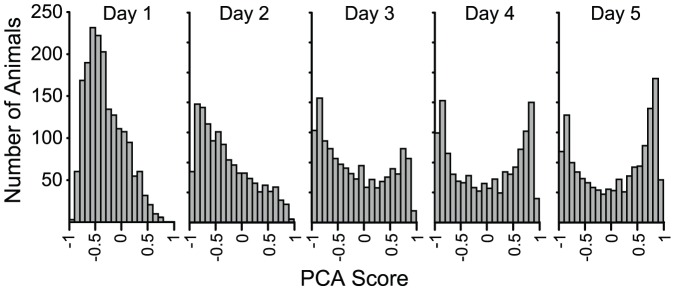
The distribution of PCA Scores across each of the 5 days of Pavlovian training, using the formula given in Table 1. The number of rats are binned according to their PCA Scores, which ranges from +1 to −1, with 0.1 bin sizes. The PCA Scores range from +1 to −1. Thus, the vertical axis shows the number of rats in each bin, and the horizontal axis the PCA Score. Note that PCA Score reveals two subpopulations of animals by Days 4 and 5 of training.

Fig. 2 shows the distributions of each of the three component measures of approach and Fig. 3 shows the distribution of the PCA Scores, on each of five days of training. It is clear from Figs. 2 and 3 that the PCA Scores and its three component measures were skewed towards negative numbers (goal-tracking) on Day 1 of training. This bias toward the goal is probably because animals were pre-trained to retrieve food from the food cup for 1–2 days prior to Pavlovian training. However, by Days 4 and 5 of Pavlovian training two subpopulations became evident. For the *Response Bias* and *Probability Difference* scores (Fig. 2), peaks associated with these subpopulations occurred at the extremes of the measurement scale, because rats that engaged exclusively in sign- or goal-tracking on all trials would get a score of +1 or −1, respectively, regardless of how many lever or magazine contacts occurred or how quickly they occurred. However, for the *Latency Score* this is not the case, because rats could respond with varying latencies even though they may have responded on every trial. The unique distribution of *Latency Score* is one rationale for its inclusion in the PCA index, as it may reflect an aspect of approach not captured by the other measures. Because two divergent subpopulations were clearly evident by Days 4–5 of training, and because the index component scores were highly correlated on these days (r's>0.90), we chose to use the average of the PCA Scores from Days 4 and 5 to produce what we call the *PCA Index Score*, and this is what is used to classify animals as STs, GTs or Intermediates (INs). Of course, other researchers could choose to classify animals based on only one component measure, which is why we provide all the data on this large sample of animals.

### Correlations between Sign-tracking, Goal-tracking and the PCA Score

**Figure 4:**
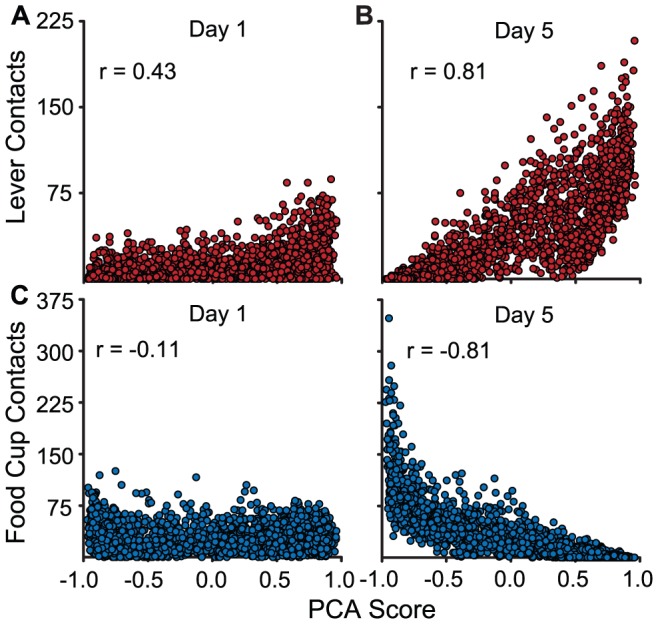
The PCA Score is strongly correlated with lever- and food cup-directed behavior on the final day of training. Selected scatterplots of correlations reported in Table 2 are shown. Each symbol represents an individual animal. The top panels show the number of lever contacts plotted as a function of the PCA Score on Day 1 (A) and Day 5 (B) of Pavlovian training. The bottom panels show the number of head entries into the food cup, during the 8 s CS period (during which time the lever was inserted into the chamber), plotted as a function of the PCA Score on Day 1 (C) and Day 5 (D) of training.

Table 2 shows correlations between the number of CS lever presses, CS food cup entries, and ITI food cup entries. On day 1, CS lever presses and CS food cup entries were weakly inversely correlated (r = −0.18), and this correlation became moderately strong by day 5 (r = −0.58). This suggests that the inverse relationship between sign- and goal-tracking increased with training. This is consistent with the segregation of individuals into subpopulations, including one that predominately engages in sign-tracking and another that predominately goal-tracks upon cue presentation. Further, although the number of lever presses was not correlated with ITI magazine entries on day 1, there was a weak inverse correlation between these measures by day 5 (r = −0.25). In contrast, a strong correlation between CS and ITI food cup entries on day 1 (r = 0.74) was weaker by day 5 (r = 0.47). These correlations suggest that this segregation of STs and GTs is not explained by differences in general exploratory behavior, as indicated by the weak associations between ITI magazine entries and sign- and goal-tracking (see also Fig. 9, and Fig. 3 in Robinson and Flagel, [9]). The PCA Score, on the other hand, as shown in Fig. 4, accounts for a substantial amount of the variance in both CS lever presses (Fig. 4A and 4B) and magazine entries (Fig. 4C and 4D) on Day 5, but less so on Day 1 of training.

**Table 2:** Correlations between Lever Contacts, CS Food Cup Entries, Inter-trial Interval (ITI) Food Cup Entries, and the PCA Scores on Day 1 (top) and Day 5 (bottom) of Pavlovian training.

Day 1	Lever Contacts	CS Food Cup Entries	ITI Food Cup Entries
Lever Contacts (1)			−0.03
CS Food Cup Entries (1)	***−0.18***		***0.74***
PCA Index	***0.43***	***−0.11***	0.00

Day 5	Lever Contacts (5)	CS Food Cup Entries (5)	ITI Food Cup Entries (5)
Lever Contacts (5)			***−0.25***
CS Food Cup Entries (5)	***−0.58***		***0.47***
PCA Index	***0.81***	***−0.81***	***−0.36***

Legend: Numbers indicate Pearson's correlation coefficient (r) and those that are Italicized numbers indicate statistically significant correlations (ps<0.01) are italicized. The number inside parentheses denotes the day of Pavlovian training.

### Analysis of Vacillation to Determine Classification Criteria

To characterize subpopulations of rats that engaged preferentially in sign- or goal-tracking on Days 4 and 5, we plotted lever- and goal-directed behavior on a trial-by-trial basis for a subset of rats (n = 370). This subset contained data from rats tested by two investigators in our laboratory (PJM and BTS). Specifically, we characterized the degree of vacillation, or switching between these behaviors, by calculating the percentage of trials in which *only* a lever press or *only* a food cup entry occurred (“ONLY” trials). Of course, it was possible for an animal to make *both* a lever deflection and a magazine entry during any given 8 sec CS period (trial). Thus, the percentage of trials in which a rat engaged in both behaviors (“BOTH” trials), and the percentage of trials in which no response occurred (“NONE” trials) was also determined.

**Figure 5:**
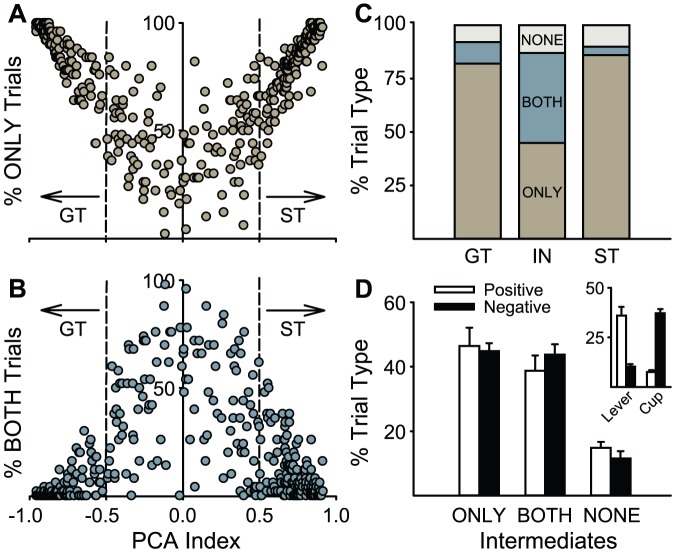
Variation in the topography of the conditioned response from trial to trial as a function of PCA Index Score. This analysis is based on a subset (n = 370) of the total sample of animals. Behavioral responses on a given trial were classed as: (1) ONLY trials (a trial in which a rat made only one or more lever deflections or only one or more food cup entries during the CS period, but not both); (2) BOTH trials (a trial in which an animal made at least one lever deflection and one food cup entry in the same 8-s CS period); (3) NONE trials (trials in which there was neither a lever deflection nor a food cup entry). Panels A and B show the percent of ONLY and BOTH trials for each rat, respectively, plotted as a function of the animal's PCA Index Score. Based on these data we classed the animals as sign-trackers (STs; a PCA Index Score of 0.5 or above), goal-trackers (GTs; a score of −0.5 or less), or intermediates (INs; scores from −0.49 to +0.49). Panel C shows the proportion of ONLY, BOTH, and NONE trials for STs, INs, and GTs. Panel D shows a more detailed analysis of the Intermediates. The INs are subdivided into those with positive vs. negative PCA Index Scores. This does not have much effect of the percent of ONLY, BOTH or NONE trials, but the inset shows that INs with positive scores (towards sign-tracking) typically press the lever on ONLY trials, and those with negative PCA Index Scores typically make food cup entries.

Fig. 5 shows the percentage of ONLY (panel A) and BOTH (Panel B) trials for individual animals, plotted as a function of their PCA Index Score (the average of the Day 4 and 5 PCA Scores). These plots indicate that rats with PCA Index Scores below −0.5 or above +0.5 had more than 50% ONLY trials and less than 50% BOTH trials. In contrast, rats with Index scores between 0.5 and −0.5 had a majority of BOTH trials and less than 50% ONLY trials. Thus, for the purpose of further analyses we chose to class animals as STs if they had a PCA Index Score from +0.5 to +1, and GTs if they had a score from −0.5 to −1. Using this criterion a ST or GT engaged in sign- or goal-tracking, respectively, twice as much as the other behavior. The remaining rats were classified as intermediates (INs; scores from −0.49 to 0.49). Note that this cut-off for classifying animals is somewhat arbitrary and more or less stringent criteria may be adopted depending on the experiment [e.g.], [ 24,28].

Using these criteria for classification, the number of BOTH, ONLY, and NONE trials for STs, INs, and GTs are shown in Fig. 5C. On average, STs and GTs performed *only* their predominant response on >80% of the trials and had relatively few BOTH and NONE trials. INs had similar numbers of BOTH and ONLY trials, which indicates that they tended to vacillate within trials as well as between trials. This is further supported by Fig. 5D, which shows the number of ONLY, BOTH, and NONE trials in INs, subdivided by whether they had positive (i.e., tendency to sign-track) or negative (i.e. tendency to goal-track) PCA Index Scores. There are no major differences in the total number of ONLY, BOTH, or NONE trials between IN rats with positive vs. negative scores. However, on ONLY trials, INs with positive PCA Index Scores most often directed their response towards the lever, whereas INs with negative PCA Index Scores typically made their response towards the food cup (insert, Fig. 5D).

### Using the PCA Index to Quantify the Distribution of Sign- and Goal-tracking Phenotypes

**Figure 6:**
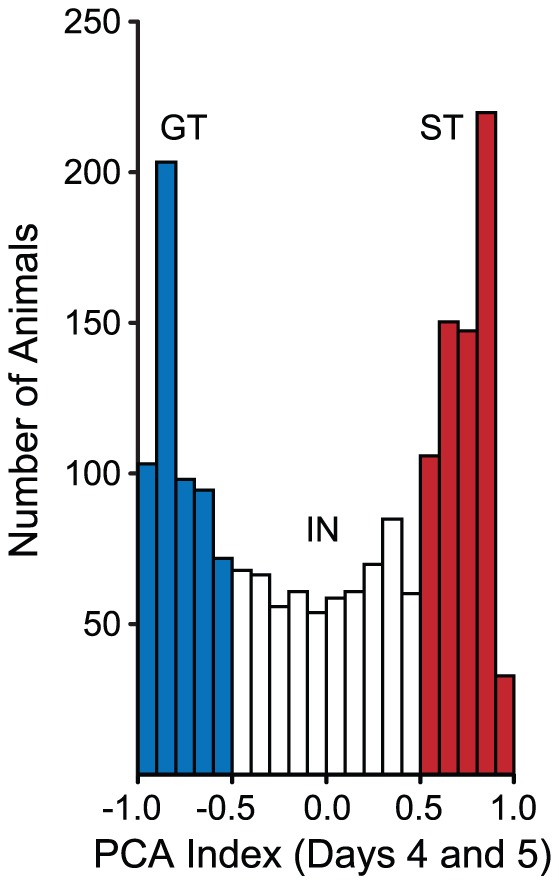
The distribution of PCA Index Scores in a sample of 1,878 rats. STs, GTs and INs are classed according to the criteria presented in Fig. 5. It is clear, based on these criteria, that STs and GTs represent different subpopulations of animals.

**Figure 7:**
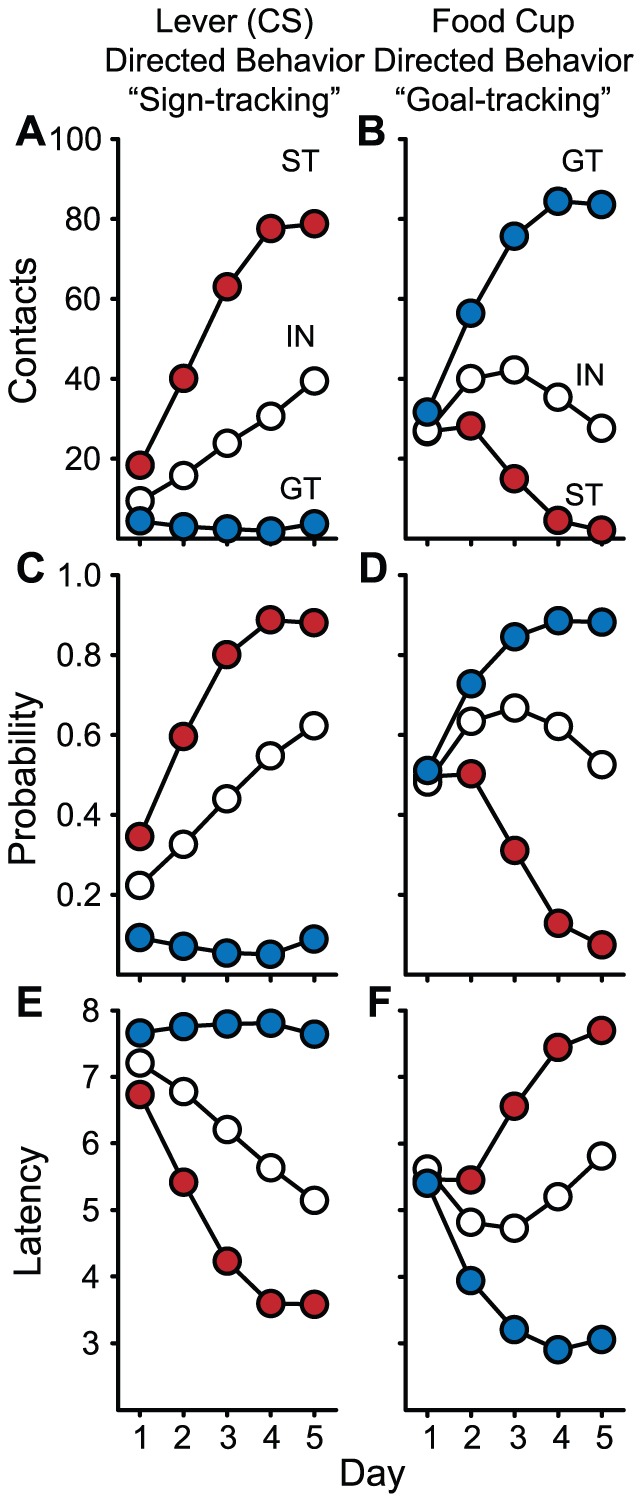
Mean ± SEM number of lever deflections (A) or food cup entries (B), probability of approaching the lever (C) or food cup (D) during the CS period, and latency to contact the lever (E) or make a food cup entry (F) during the CS period, over 5 days of Pavlovian training in 1,878 rats classed as STs, GTs or INs, as described in Fig. 6. Note that the SEM is smaller than the symbol in most cases.

Next, we calculated PCA Index Scores for all the 1,878 rats in our sample, and using the criteria described above, classed them as STs, GTs or INs. Using this criterion, and this relatively large sample of the population, we found that 657 (35%) were STs, 650 (35%) were IN, and 571 (30%) were GTs (Fig. 6). The behavior of rats classed in this way across the 5 days of training is shown in Fig. 7. As expected, there were robust differences in all three measures of Pavlovian conditioned approach behavior, with STs showing increases in the measures of sign-tracking concomitant with a decrease in the goal-tracking measures. The reverse was true for GTs, while INs displayed intermediate values.

**Figure 8:**
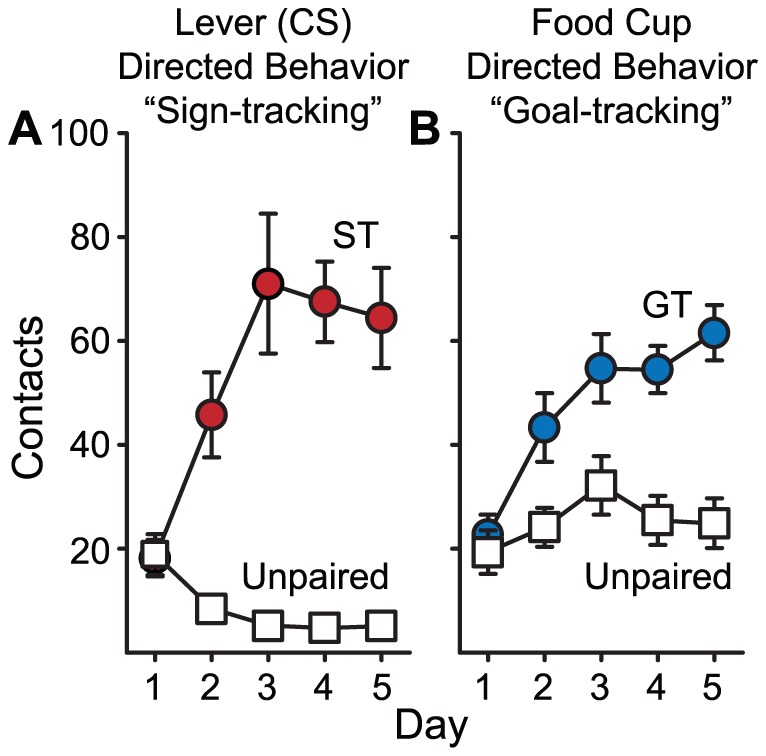
Animals in which the CS and US are presented but not paired do not learn either a ST or GT CR. (A) The mean (± SEM) number of lever presses in STs and the Unpaired group (UN) and (B) CS food cup entries in GTs and the Unpaired group. The data are reanalyzed from Robinson & Flagel [9], and STs and GTs were re-classed based on the PCA Index.

**Figure 9:**
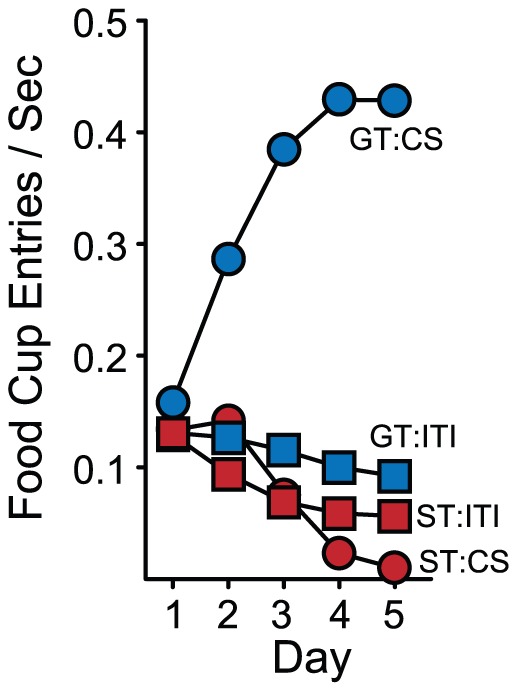
Mean (SEMs are occluded by the symbols) frequency of food cup entries (entries/s) during the CS period vs. intertrial (ITI) periods in sign-trackers (STs) and goal-trackers (GTs) over five days of Pavlovian training. The STs and GTs are those shown in Fig. 6 and 7. The data illustrate that GTs discriminate CS vs. non-CS periods, selectively increasing head entries during the CS period as a function of Pavlovian training.

It is important to note that learning either a ST CR or GT CR requires CS-US pairings. Animals for which the CS and US are presented in an unpaired fashion (n = 51), do not learn either CR (Fig. 8), as reported previously [9], [15], [25], [27], [34]. However, this does not address whether goal-tracking is associated with a general increase in magazine entries, even during the ITI periods. We determined whether GTs discriminated between the CS and non-CS periods by comparing head entries made by STs and GTs during the non-CS period (when the lever was not extended). Fig. 9 shows the frequency of head entries during the CS and non-CS periods in STs and GTs. GTs entered the food cup more frequently than STs during the non-CS period. However, GTs clearly discriminated the CS and non-CS periods, evidenced by a decrease in the non-CS entries across days of training concurrent with a large increase in CS entries. Also, by the end of training, GTs make head entries much more frequently during the CS period than during the non-CS period.

### Predicting Conditioned Reinforcement with the PCA Index

**Figure 10:**
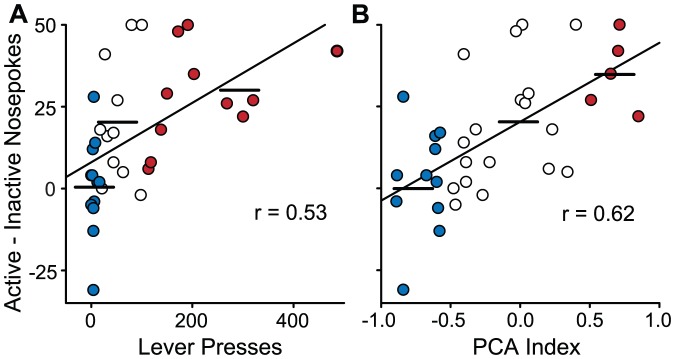
The propensity to approach a lever-CS predicts the ability of the same lever-CS to support learning a new instrumental response to get it (i.e., the ability of the lever-CS to act as a conditioned reinforcer). Data from Lomanowska et al. [25] were used to compare the effectiveness of the rank-order split and PCA Index methods to predict the ability of the CS to act as a conditioned reinforcer. For the rank-order split method, rats were classed as STs and GTs by totaling the number of lever contacts over 5 days of Pavlovian training and dividing the sample of animals tested into thirds. Panel A shows the correlation between active nose-pokes (minus inactive nose-pokes) on the test for conditioned reinforcement, as a function of total lever contacts. Panel B shows the same data, but when each animal's PCA Index Score was calculated and used to class animals. In both Panels red filled symbols indicate GTs, white symbols INs, and blue filled symbols STs, classed the two different ways. Horizontal lines depict group means. (Note that the sample sizes differ for the groups between the two methods; an equal number of STs and GTs cannot be assumed when using the PCA Index.).

To further test the effectiveness of the PCA Index method to predict the propensity of animals to attribute incentive salience to reward cues we determined how well it predicts individual variation in this trait when it is assessed using a different behavioral measure of incentive salience attribution. Robinson and Flagel [9] first reported that a lever-CS serves as a better conditioned reinforcer in STs than GTs, and this effect has now been reported in two additional studies [25], [34]. Data from Lomanowska et al. [25] were used to compare the effectiveness of the rank-order split and PCA Index methods to predict the ability of the CS to act as a conditioned reinforcer. For the rank-order split method, STs, INs, and GTs were classed by totaling the number of lever contacts over 5 days of Pavlovian training and dividing the sample of animals tested into thirds, as described previously [9], [32]. We compared this method with the PCA Index described here (Table 1, Fig. 6). Fig. 10 shows that the correlation between conditioned approach and conditioned reinforcement obtained using the rank-order split method (Fig. 10A; r = 0.53; p<0.01) was not quite as strong as when the PCA Index Scores were used (Fig. 10B; r = 0.62; p<0.001). This indicates that the PCA Index classification method is an effective predictor of conditioned reinforcement. In further support, we also reclassified rats used in the Robinson and Flagel study (see Fig. 3 in [9]) using the PCA Index, and found a very similar correlation between the PCA Index Scores and the measure of conditioned reinforcement (r = 0.64; p<0.001; data not shown), replicating the results shown in Fig. 10. In summary, the PCA Index strongly predicts the tendency to attribute incentive salience to a food cue, even when assessed using a completely different measure of incentive salience attribution (i.e., conditioned reinforcement), and in an instrumental rather than Pavlovian setting.

## Discussion

The idea that the incentive motivational properties of reward-associated cues are especially important in controlling behavior has a long history [2], [4], [12], [13], [35], [36], [37], [38], [39], [40], [41], [42], [43]. However, in many studies examining the control of behavior by reward cues it is often assumed (either explicitly or implicitly) that the conditional relationship between a stimulus (CS) and a reward (US) is sufficient to confer incentive motivational properties to the CS. That is, if a CS is capable of evoking a conditioned response (CR) it also has the ability to act as an incentive stimulus. We suggest this is not the case.

The data presented here show that, in fact, there is considerable individual variation in the extent to which a perfectly effective CS acquires the properties of an incentive stimulus. We report, using a large sample of the population (N = 1,878 rats), that a food cue becomes powerfully attractive, reliably eliciting approach towards it, in only about 35% of animals. That is, based on a measure of one property of an incentive stimulus (attraction/approach), only a subset of the population attributed the food cue with sufficient incentive salience for it to become powerfully attractive. Importantly, the PCA Index Score predicts the extent to which a food cue becomes itself desired (another property of an incentive stimulus), based on whether animals will perform a new instrumental response to get it (i.e., to act as a conditioned reinforcer; Fig. 9 and [9], [25], [34]). The correlation between the PCA Index Score and conditioned reinforcement ranged from r = +0.62 to +0.64 in data reanalyzed from two independent studies [9], [25]. A similar association between PCA and conditioned reinforcement has been reported in two selectively-bred lines of rats that differ markedly in their propensity to approach a food cue [34]. We have also reported that a food cue is more effective in reinstating food-seeking behavior after extinction of the instrumental response in rats that find the food cue attractive (STs), relative to rats that are not attracted to a food cue (GTs) [26].

We suggest, therefore, that the predictive value of a CS, required for it to evoke a CR, is not sufficient to confer incentive value. That is, a CS is not necessarily also an incentive stimulus. The attribution of incentive salience to a reward cue, which is necessary for it to acquire Pavlovian conditioned motivational properties, requires additional processes [10], [34], [44], [45], [46]. This is an important distinction, because only if reward cues acquire incentive motivational properties will they also acquire the ability to exert strong control over motivated behavior, and thus the ability to potentially instigate maladaptive behavior.

This notion is further supported by reports that the attractiveness of a food cue predicts the extent to which a drug (cocaine) cue acquires incentive motivational properties, based on a number of different measures [27], [29], [30], [31], [47]. Thus, relative to GTs, STs are more likely to approach a cocaine cue [27], [28], they work harder for cocaine [29], and show greater cue-induced and cocaine-primed reinstatement following extinction of self-administration behavior [29], [30]. Furthermore, only STs develop a preference for a cocaine-associated tactile cue in a conditioned cue preference procedure, and emit frequency-modulated 50 KHz ultrasonic vocalizations in the presence of the cocaine cue [31]. Finally, a cocaine cue is more important in maintaining cocaine self-administration behavior in STs than GTs [30], [48].

It is important to emphasize that *both* the ST CR and GT CR are learned responses, acquired with experience, and both STs and GTs learn their respective CRs at a comparable rate (Fig. 7 and Fig. 9). For both STs and GTs, pairing the CS and US increased the probability of approach during the CS period, to either the cue or the goal, respectively. It increased the vigor with which they engaged the cue or the goal, and it increased the rapidity with which they approached their respective targets upon CS onset (Fig. 7). In addition, if the food cue is *not* explicitly paired with food delivery, rats fail to learn either a ST or GT CR (Fig. 8; [9], [25], [27]). Furthermore, we have trained animals for up to 22 days and the ST, GT and intermediate phenotypes remain dissociated and stable [9]. Finally, STs and GTs do not differ systematically in learning other food-reinforced tasks [9], [26]. Therefore, it is clear that the cue (CS) acts as a predictor, providing the information necessary to support learning the CS-US association equally in STs and GTs. We suggest that the difference in the topography of the CR reflects the extent to which the CS is attributed with incentive salience (see below for more discussion).

### Advantages of the PCA Index Score in Estimating Individual Variation in the Propensity to Attribute Incentive Salience to Reward Cues

Incentive stimuli are defined by their ability to attract, become ‘wanted’, and generate a conditioned motivational state [4], as assessed using PCA, conditioned reinforcement, and Pavlovian-to-Instrumental transfer (PIT) tests [17], [49], [50], [51]. Although these features collectively define an incentive stimulus, it is important to note that they are themselves psychologically and neurobiologically dissociable [e.g.], [ 39], [52], [53,54]. Nevertheless, in order to estimate the propensity to attribute incentive salience to reward cues, we have focused on PCA rather than conditioned reinforcement or PIT, for three reasons. First, PCA is procedurally and psychologically less complex. For example, in order to measure conditioned reinforcement or PIT, individuals must first undergo Pavlovian training anyway, which may obviate the need for additional tests. Also, PCA does not involve discrimination or instrumental learning, as with the other tasks. Second, for assessing PIT, auditory cues are usually used, but these elicit only goal-tracking [55], [56], [57] and do not acquire incentive properties differentially in STs and GTs [58]. Localizable visual cues are problematic in typical PIT experiments because such cues can elicit sign-tracking, pulling an animal away from the instrumental manipulandum, thus interfering with transfer. Finally, conditioned reinforcement and PIT effects are often transient and fragile, which may be because these tests are conducted in the absence of reward and therefore are subject to extinction processes.

In a number of previous studies we [9], [25], [26], [27], [30], [31], [32], [33], [58] and others [59] have classed rats as STs or GTs based on the number of lever contacts alone, typically dividing relatively small samples of the population into thirds based on the total number of lever contacts over five days of training (the “rank-order split” method). With this procedure it is assumed that the tendency to sign-track is inversely related to the tendency to goal-track. This is partially true: sign-tracking and goal-tracking are indeed inversely correlated (Table 2), but this is not a perfect correlation (r = −0.58). The rank-order split method would presumably identify rats that did not interact with the lever, but a rat that made 15 lever contacts and 100 CS magazine entries during a session would be ranked the same as a rat who made 15 lever contacts and 0 magazine entries. Making each of the Index components a subtraction score, in which each measure of goal-tracking is subtracted from the related measure of sign-tracking, solves this problem and places equal emphasis on sign- and goal-tracking.

Another major advantage of using the PCA Index Score developed here in a very large sample is that small groups, or even single animals, can be classed as a ST, IN, or GT, as long as similar training procedures are used. This can be helpful for studies with low samples sizes, like many neurobiological studies. This would not be possible with the rank-order split method. Furthermore, the PCA Index is insensitive to differences between samples. For example, a given small group of rats arriving from the supplier could, by chance, all have PCA Index Scores above 0.5, and the rank-order split method would erroneously identify some of these rats as GTs. Indeed, we have found there is sometimes considerable variation in the prevalence of STs vs. GTs, both between batches of rats purchased from the same supplier, and between batches purchased from different suppliers. These effects may be dependent on the breeding and selection practices of commercial suppliers as well as differential allelic frequency changes within isolated populations. It could also be due to differences in how animals are handled prior to shipment, or during shipment, or to when the rats were weaned (which may vary considerably; M. Marinelli, personal communication), as early life experience is known to influence the propensity to show sign-tracking vs. goal-tracking in adulthood [25], [60]. Therefore, the PCA Index method increases reliability by allowing the comparison of an individual's performance relative to a large sample of the population.

At first glance, it may seem redundant to include three highly correlated measures for calculation of the PCA Index. Response bias, probability difference, and latency difference are strongly correlated on Day 5 (r's>0.93). For example, rats that contact the lever quickly (short latency) are likely to have a greater response bias for the lever (many overall lever contacts). We included all three measures of approach so that, when PCA is used as a classification process, the PCA index would be less sensitive to random fluctuations in one of the measures. However, when PCA is the endpoint of an experiment, certain experimental manipulations may alter one of these measurements differentially. In this case, the three measure of approach should be examined independently. For example, some rats may engage the lever only briefly, immediately after CS onset, while engaging the food cup for the remainder of the CS period. In this manner, response bias would be more sensitive to the goal-tracking aspect of the behavior, and would thus be dissociated from latency to food cup entry and probability. An experimental treatment that resulted in an increase in vacillation between sign- and goal-tracking could produce such a dissociation. For example, lateral striatal lesions produce response-bias alterations with no effect on response latency in a visual discrimination task; the reverse is true for medial striatal lesions [61]. This suggests that latency and response bias in general are subserved by distinct neurobiological processes, and this may be true for conditioned approach as well. In addition, non-food USs, such as drugs or rewarding electrical brain stimulation, may produce rapid approach, but very little physical interaction with a lever-CS [62], [63], [64], [65]. The PCA Index would be sensitive to changes such as these, although it would need to be adapted to measure approach to the cue, independent of physically engaging it (e.g., using video-based proximity analysis). Lastly, the application of the PCA Index can be used to compare more accurately the results across studies and laboratories where procedures may vary. For example, by using the PCA Index, studies using variable number of trials per session (e.g., 25 vs. 40), variable CS durations (e.g., 5s vs. 8s vs. 10s) or even more days of training can be more accurately compared. Previous approaches emphasizing raw numbers of CS contacts and magazine entries are difficult to compare if the number of trials or other variables are not consistent across studies.

### State versus Trait?

We have suggested that the PCA Index is useful in assessing a complex psychological trait, which we have defined as *the propensity to attribute incentive salience to discrete, localizable reward cues*, and that it indicates that there are two major behavioral phenotypes: STs, which refers to animals prone to attribute incentive salience to localizable reward cues, and GTs, which are less prone to do so.

We define propensity as an inclination or natural tendency to behave in a certain way, and we suggest that this propensity is consistent across situations. However, that this is only a tendency underlines that there are situations in which the individual differences in this propensity may not be evident. An important issue to consider, therefore, is whether the ST/GT distinction revealed by the PCA Index described here does in fact represent a stable complex psychological trait, or only transitory “states” expressed under specific conditions. First, it is critical to emphasize that the psychological trait of interest is *not* sign-tracking or goal-tracking behavior *per se*. Rather, a ST CR and GT CR represent only two of a number of possible behavioral measures associated with the underlying psychological trait. We use the terms “STs” and “GTs” as a short-hand to refer to individuals that not only differ in attraction to a reward cue, but also show other behavioral differences indicative of incentive salience attribution to cues, including those assessed with measures of conditioned reinforcement and conditioned motivation [4], [10], [11], [17], [49], [50], [51]. We assume this trait, like all complex psychological traits, is determined by gene X environment interactions, although the genetic or epigenetic basis of the trait and the critical environmental determinants are unknown [although see 60], [66], [67]. Below we summarize the evidence that STs and GTs represent individuals that express two different behavioral phenotypes.

#### 1. The two phenotypes are heritable

In rat lines selectively bred for their locomotor response to a novel environment, the propensity to attribute incentive salience to reward cues (as assessed with multiple measures on multiple tests — not just ST behavior) also segregated [27], [34]. That the HR/LR and ST/GT traits co-segregated suggests a partial genetic overlap at best, because there is no relationship between these two phenotypes in outbred rats [9], [48]. Nevertheless, the fact ST/GT was subject to selection across generations is evidence for heritability. Further, these studies confirmed that the ST/GT phenotype is associated with behavioral responses in drug-conditioning paradigms and in measures of impulsivity [27]. In these studies, the animals were not trained in the PCA paradigm but were identified as STs and GTs based on their breeding history. Therefore, these phenotypes are not a consequence of PCA training itself [34].

#### 2. It is modified by early experience

We have shown that early life (preweaning) adversity increases the proportion of STs in the population [25]. Complementary to this finding is the report by Beckmann & Bardo [60] showing that post-weaning isolation also increases the likelihood of sign-tracking while post-weaning rearing in complex (‘enriched’) environment increases the probability of goal-tracking. Thus, these findings suggest a gene X environment interaction, as is typical for complex psychological traits.

#### 3. The phenotypes are stable and predictive

As we report here, and elsewhere [9], [25], performance on one task (e.g., a ST CR) reliably predicts performance on others indicative of the trait, assessed at a different time and using very different procedures (Fig. 10). The propensity to approach reward cues not only predicts the ability of a cue to act as conditioned reinforcer [9], and to reinstate behavior [26], but also the behavioral responses to very different cues (e.g., a cocaine cue) that have acquired incentive value in an instrumental, rather than a Pavlovian setting [29], [30]. ST behavior even predicts responses to a fear cue [68]. Also, a ST CR is stable over at least 22 days of testing [9], and is intact after six weeks without additional PCA training (PJM, unpublished data). There is considerable evidence, therefore, that expression of the traits is not idiosyncratic to one testing situation - it is manifest in many different tests.

#### 4. The two phenotypes are associated with biological differences

This requires much more work but we have identified differences between STs and GTs in dopamine systems and HPA axis responsivity, and different patterns of immediate-early gene expression in cortical-striatal-thalamic brain regions [14], [32], [34], [69].

#### 5. The two phenotypes are associated with other traits in a predictable and logical way

STs tend to be impulsive on tests of impulsive action, but interestingly, not on tests of impulsive choice [27], [66]. In addition, Beckmann et al. [48] have reported that STs also tend to show greater *novelty-seeking* behavior. It is worth noting, however, that although significantly related, variance on a test of impulsive action only accounts for about 15% of the variance on a test of attraction to a food cue. This means some STs will be impulsive, but others may not. It may be that it will be those individuals who are prone to attribute incentive salience to reward cues, *and* have poor top-down cognitive control (resulting in impulsivity), *and* are novelty-seekers, who will be especially vulnerable to develop impulse-control disorders, such as addiction [70], [71].

6. Similar behavioral propensities are seen in humans [72] and are related to genetic variants [73], [74], [75].

Of course, we readily acknowledge that whether sign-tracking or goal-tracking behavior is expressed in a given context is strongly influenced by situational factors [15], [17]. Indeed, the context can be structured so that all animals develop a GT CR. For example, rats generally will not learn to approach an auditory cue paired with food delivery [58], [76]. Thus, if a tone is used as the CS, all rats learn a GT CR and the tone-CS is equally effective as a conditioned reinforcer in STs and GTs [58]. This indicates that GTs are not just generally impaired in their ability to attribute incentive salience to reward cues, as they do so perfectly fine in some situations. However, if a situation is structured such that STs do not show ST behavior (e.g., using auditory cues that are difficult to localize) this does not mean that the underlying trait is not present or stable. Conversely, the PCA paradigm could be altered to enhance the prevalence of sign-tracking, thereby creating a state in which a majority of subjects attribute significant incentive value to a specific cue. But this does not necessarily alter the underlying trait. In other words, environmental factors may determine whether any given trait is expressed at any given time or in any given situation, as is the case for all complex psychological traits.

### What are the Psychological Processes Involved in Sign- and Goal-tracking?

Sign- and goal-tracking are both forms of appetitive learning, but recent neurobiological studies suggest that they are subserved by different neural systems, and therefore, presumably different psychological processes. What might these processes be?

While reward expectation may play a role in the goal-tracking response (see next paragraph), a non-reinforced behavior such sign-tracking cannot be due to expectancy alone [5], [35], [58]. In addition, sign-tracking is not a case of simple stimulus substitution, because it depends on the nature of the cue, the status of the reward, and the physiological drive state [77], [78], [79], [80], [81]. The available evidence suggests that sign-tracking is a behavioral manifestation of Pavlovian incentive motivational processes, which occur as a result of a multiplicative interaction between the transfer of incentive value to the cue and the physiological drive state [2], [11], [12], [44], [46], [82], [83]. That incentive for cues and their associated rewards is dissociable is supported by neurobiological investigations of sign-tracking. For example, the learning and expression of sign-tracking (but not goal-tracking) are blocked by dopamine antagonists [34], [84], [85], [86]. In addition, sign-tracking (but not goal-tracking) is associated with the transfer of a phasic dopamine signal from the US to the CS in the nucleus accumbens [34]. This involvement of the mesolimbic dopamine system is consistent with sign-tracking reflecting the output of a bottom-up, unconscious motivational process that is associated with impulsivity and poorly controlled by top-down cognitive processes, which may involve corticostriatal projections [66], [87], [88].

Toates [12] has suggested that Pavlovian incentive processes can occur simultaneously alongside cognitive processes resulting in goal-directed behaviors, such as goal-tracking. At first glance, it may seem that goal-tracking is also dependent on dopamine, because others have reported that dopamine antagonists block the goal-tracking response in a PCA paradigm [55], [56]. However, these studies measured the response to an auditory cue, as opposed to visual cues used in studies of sign-tracking. This is a key consideration, because while goal-tracking in response to an auditory cue appears to be dependent on dopamine, goal-tracking in response to a discrete visual cue is not [34], [84], [89]. In addition, when trained with a compound visual/auditory stimulus, the auditory component induced goal-tracking in both STs and GTs, and was also an equally effective conditioned reinforcer for both. In contrast, the visual component was much more effective in sign-trackers [58]. Therefore, learning to goal-track in response to a visual cue is unique in that it is dopamine independent. However, there is little information from studies of Pavlovian incentive learning regarding the psychological and neurobiological substrates of this goal-tracking CR.

Studies of *instrumental* incentive learning may provide some clues. Dickinson [90] used the term “act-outcome” learning to suggest that individuals have a cognitive understanding of the results of their actions. In this sense, behavior is mediated by the explicit expectation of the outcome [91], [92]. This form of act-outcome learning requires a cortico-striatal system that is distinct from the mesolimbic dopamine system involved in Pavlovian incentive learning [55], [93], [94]. Further, as Toates [12] suggested, expectancy-based and Pavlovian forms of incentive learning can occur in tandem. For example, studies by Corbit, Balleine, and colleagues [95], [96], [97] have examined the role of several brain areas in two varieties of PIT. In the “general” form, a Pavlovian CS energized responding for a multiple food rewards. In the “outcome-specific” form, the CS only energized responding for the same reward that it was paired with. Only the “general” form is affected by a shift from hunger to satiation [95], and was dependent on the nucleus accumbens core and the central nucleus of the amygdala [96], [97], which are two critical areas involved in sign-tracking [84], [86], [98]. Therefore, sign- and goal-tracking may be analogous to the processes engaged by the general and outcome-specific forms of PIT, respectively, which is paralleled by their differential dependence of mesolimbic dopamine. If true, then this would indicate that goal-tracking is a function of a stimulus-outcome expectancy that involves cortical areas such as the prefrontal and insular cortices [99]. This possibility is supported indirectly by two recent studies showing that 1) c-fos mRNA expression was correlated in the cortico-striatal circuit in GTs but not STs [24], and 2) goal-trackers display better attentional control during a sustained attention task; performance in this task is controlled by cholinergic systems within the prefrontal cortex and basal forebrain [87], [100]. Further, this dissociation between Pavlovian incentive salience and stimulus-outcome expectancies may be paralleled by different computational constructs of incentive salience attribution [which may describe sign]-[tracking; 44,45,101]. Future studies are clearly required to test these hypotheses.

### The Irresistibility of Reward Cues Can Lead to Maladaptive Behavior

One of the most remarkable features of sign-tracking is that reward-related cues can become so irresistibly attractive that they produce seemingly maladaptive and arguably compulsive behavior that delays or leads to the loss of the primary reward [17], [20], [102], [103], [104]. For example, Hearst and Jenkins [17], tested pigeons in a long box in which a key light on one end of the box preceded delivery of food at the other end, and the pigeons would traverse the length of the box and peck on the key light although this had no effect on the probability of food delivery. Most remarkably, if the food were only available for a short period of time, such that walking to the far end of the box would result in loss of the reward, the pigeons continued to peck the key light. Another example comes from studies using an “omission schedule”, whereby contact with the CS results in omission of the reward. Despite the potential loss of reward, animals will continue to approach and sometimes contact the CS [104]. A final example comes from studies in quail in which a terrycloth-covered object was used as the CS and presentation of a female (to male quail) as the US. With training some male quail came to approach, mount and even copulate with the terrycloth object, and some of these, “were often observed to continue copulating with the CS even after the female was released” [105]. The CS acquired such powerful incentive motivational properties that it was preferred over a real female! These examples vividly demonstrate just how irresistibly attractive incentive stimuli can become, and how powerfully they can control behavior. They also indicate that approach behavior is *not* maintained by “accidental reinforcement” of an action, or “superstitious” behavior [15], [20], [106], [107], [108].

Given the apparently maladaptive nature of sign-tracking behavior in these laboratory situations one can ask why such a large proportion of animals develop this response. Presumably, the answer is that in environments in which animals evolved, sign-tracking behavior is often adaptive. Stimuli in the environment that are associated with reward delivery or location help organisms obtain rewards such as food. In most situations, cues predictive of reward would be located at the same place where the reward itself is to be found. A strong tendency to approach such cues (sign-track) would bring the animal into close proximity of the food, and increase the probability of obtaining it, even if the food were concealed. For example, an effective strategy for a hungry bird looking for a worm would be to peck a worm-hole, even if the worm is not immediately visible. Rapidly approaching reward cues would also increase the probability that a given animal, rather than another individual competing for similar resources, would get the reward. Thus, in many contexts (but presumably not all) sign-tracking would be an adaptive strategy [22], [109], [110], [111]. It could be argued that sign-tracking only leads to maladaptive behavior in laboratory situations when the cue is deliberately located at a place some distance from the location of reward delivery. However, in humans, it may also lead to maladaptive behavior in modern environments, where an abundance of signs (cues) predict the availability of inordinately large amounts of rich, high fat and/or sugary foods. It may also lead to maladaptive behavior when cues predict inordinately powerful rewards, such as drugs.

The idea that incentive stimuli are important in controlling behavior in humans, and may contribute to maladaptive behavior, is well established [112], [113], [114], [115], [116], [117], [118]. For example, there are many studies showing that food-associated cues can evoke desire for food, and this is thought to contribute to some eating disorders [119], [120]. Indeed, Beaver et al. [73] report that individuals who score high on a “Behavioral Activation Scale (BAS)”, thought to assess the propensity for appetitive motivation, “experience more frequent and intense food cravings and are more likely to be overweight or develop eating disorders associated with excessive food intake”, and this is associated with greater activation of mesocorticostriatal circuits in response to images of food. Indeed, there are numerous similarities in the brain regions activated by food, sexual, and drug cues [118], [121], [122].

In the case of drugs, it is well-established that drug-users are attracted to drug cues [70], [123], their attention is biased towards them [71], [124], [125], [126] and they preferentially choose them [114]. In cocaine dependent subjects the extent to which cocaine cues disrupted performance in the Stroop Task even predicted treatment outcomes [127]. Drug cues also support responding on a second order schedule of reinforcement in humans similar to that observed in non-human animals [128]. Many studies have established that drug cues can evoke craving and/or relapse [115], [129], [130]. Although there has been some debate concerning the relationship between craving and relapse, a recent study of drug users in their normal living environment found that, “cocaine craving is tightly coupled to cocaine use” [131]. Interestingly, Mahler and de Wit [72] recently reported that smokers who showed high craving when presented with food cues when hungry also showed the highest craving when presented with smoking cues after a period of abstinence from cigarettes.

In addicts, drug cues activate mesocorticolimbic circuits known to be important in mediating the incentive motivational properties of reward cues [132], [133], [134]. Indeed, even words related to drug use are sufficient to activate brain motive circuits [135], as are “unseen cues” [118]. The latter finding is interesting because it suggests implicit drug cues, outside of conscious awareness, are sufficient to activate brain motive circuits. This is important because, as put by Childress et al. [118]:

*“By the time the motivational state is experienced and labeled as conscious desire, the ancient limbic reward circuitry already has a running start. This dilemma may be reflected not only our daily human struggle to manage the pull of natural rewards such as food and sex, but also in the chronic, treatment resistant disorders for which poorly controlled desire is a cardinal feature (e.g., the addictions).”*

Finally, there is accumulating evidence for considerable individual variation in the ability of cues to evoke motivational states and activate mesocorticolimbic circuitry in humans, some of which is due genetic variation [73], [74], [75], [136], [137]. It will be important to determine if individual variation in the propensity to attribute incentive salience to reward cues, as described here in rats, is biologically related to similar variation in humans.

## Materials and Methods

### Subjects

1,878 adult male Sprague Dawley rats (200–300 g) were purchased from Harlan or Charles River. Rats were handled daily during the week leading up to testing and were given ∼25 banana-flavored pellets (45 mg, BioServ; Frenchtown, NJ) in their home cages (to familiarize them with the pellets) for two days prior to testing. The rats were tested by six different laboratory members from Terry Robinson's laboratory over the course of six years (2004–2010). In addition, the data shown in Fig. 10 were collected by Anna Lomanowska and Vedran Lovic at the University of Toronto – Mississauga; these rats were purchased from Charles River. Otherwise, rats were treated identically as described below. Data from previously published studies are included in the analysis reported here [9], [24], [25], [26], [29], [30], [31], [32], [33], [66], [68], [69], as well as data from unpublished studies. Rats were given free access to food and water when not in the conditioning chambers (i.e., they were not food restricted). All experiments followed the principles of laboratory animals care specified by “Guidelines for the Care and Use of Mammals in Neuroscience and Behavioral Research” National Research Council (2003), and all procedures were approved by the University Committee on the Use and Care of Animals at the University of Michigan.

### Apparatus

Conditioning chambers (20.5×24.1 cm floor area, 29.2 cm high; MED-Associates Inc., St. Albans, VT) were situated in sound attenuating cubicles outfitted with ventilation fans. Each chamber contained a red house light centered near the ceiling of one wall. A food magazine was centered on the opposite wall and was flanked, either to the left or right, by a retractable lever. When the lever was inserted into the chamber the slot through which it protruded could be illuminated by activating a LED mounted behind the wall. A catch tray filled with corn-cob bedding was located underneath the floor, which was constructed from stainless steel rods. Chambers were controlled by MED-PC software.

### Pretraining

In our earlier studies pretraining consisted of two daily sessions in which rats were placed into the chamber for five minutes, after which time the red house light was illuminated and 50 banana-flavored food pellets were delivered on a Variable Time (VT) 30 s schedule (i.e., one pellet was delivered on average every 30 s, but the exact time varied randomly between 1–60 s). The lever was always retracted during these sessions. Rats were returned to their home cages immediately after the session. Rats that did not eat all the pellets by the end of the second session, which occurred in <1% of rats, were not tested further. In later studies we found that this pretraining period could be reduced to a single session in which the rats received 25 pellets instead of 50, so some animals were pretrained using this schedule. This was sufficient to familiarize them with the magazine (it was very rare that rats did not eat all the pellets during this phase). Approximately 1,000 rats were tested using the 2-day procedure while the remainder were tested using the 1-day procedure.

### Pavlovian Conditioning Procedure

On the day following the pretraining session(s), rats underwent five daily sessions of Pavlovian training. One minute after rats were placed into the chamber, illumination of the red house light signaled the beginning of the session, and the house light was left on throughout the entire session. The lever was inserted into the chamber for 8 s, and during this time the LED located behind the lever was illuminated. After 8 s the lever was retracted, the light extinguished, and a food pellet was immediately delivered into the adjacent food cup. Each training session consisted of twenty-five lever-pellet pairings using a VT-90 s schedule (i.e., presentation of the CS and US varied randomly between 30–150 s, with an average of 90 s). Each session lasted, on average, 37.5 min. Lever presses were recorded when the rats deflected the lever, and food cup entries were recorded as interruption of a photobeam across the entrance to the food cup. Note that pellet delivery occurred independent of the animal's behavior. Rats were returned to their home cages at the end of the session.

### Conditioned Reinforcement Procedure

Some rats were also subjected to a 40-min test for conditioned reinforcement. The data from these tests have been reported previously [9], [25], but are reanalyzed here. On the day after Pavlovian training, as described above, rats were placed into the conditioning chambers, but these were reconfigured. The food magazine was removed and the lever was now positioned in its place, in the center of the wall. Two nose-poke ports (2 cm diameter; 2 cm above the floor), equipped with photocells, were added, one on each side of the lever. One port was designated “Active” and the other “Inactive”. When a rat made a nose poke into the Active port the lever was extended into the chamber (and illuminated) for 2–4 s. Nose pokes into the Inactive port had no consequence This test occurred under extinction conditions, in that no food pellets were delivered. The number of responses into the ‘Active’ and ‘Inactive’ ports were recorded.

### Data Analysis/Statistics

Lever deflections and magazine entries were recorded during the CS and ITI periods for the five days of training. The values were used to calculate the Response Bias, Latency Score; Probability Difference, and PCA Index (see Results, Table 1). Descriptive statistics (means, frequency histograms, scatterplots) were generated using Statistica (Tulsa, OK). Pearson's product moment correlations were calculated for the correlations presented in Table 2 and Fig. 10.
